# Outcomes of music therapy on children and adolescents with attention-deficit/hyperactivity disorder: a systematic review and meta-analysis

**DOI:** 10.47626/2237-6089-2024-1020

**Published:** 2025-12-05

**Authors:** Adriana de Oliveira Goes, Antonio Egidio Nardi, Laiana Azevedo Quagliato

**Affiliations:** 1 Universidade Federal do Rio de Janeiro Instituto de Psiquiatria Laboratório de Psiquiatria da Infância e Adolescência Rio de Janeiro RJ Brazil Laboratório de Psiquiatria da Infância e Adolescência, Instituto de Psiquiatria, Universidade Federal do Rio de Janeiro, Rio de Janeiro, RJ, Brazil.

**Keywords:** ADHD, attention deficit hyperactivity disorder, music therapy

## Abstract

**Objective:**

Attention-deficit/hyperactivity disorder (ADHD) affects approximately 7% of children and 5% of adolescents worldwide. ADHD is characterized by inattention, hyperactivity and impulsivity as symptoms. The exact mechanism underlying this disorder is still unclear, but genetic and environmental factors play important roles. In recent years, research and treatment options have increased, including medication and/or therapeutic interventions such as music therapy. There is evidence showing benefits of music therapy on mental health, such as autism spectrum disorder (ASD). However, results regarding ADHD are limited. The aim of this meta-analysis was to verify whether music therapy is an effective tool for improving symptoms in children and adolescents with ADHD.

**Methods:**

In accordance with PRISMA guidelines, we systematically searched the Virtual Health Library, SciELO, PubMed and Cochrane Library databases. This article included original research that investigated the effect of music therapy on hyperactive and impulsive symptoms in children and adolescents with ADHD.

**Results:**

Music therapy was associated with a trend of efficacy involving ADHD symptoms (effect size: 1.18; CI: −3.8 – 0.21; p = 0.08). Additionally, a significant amount of heterogeneity among trials was found (I2 = 92 %). The lack of studies involving music therapy, ADHD and brain imaging, as well as limited investigation of inattention symptoms in studied articles are limitations of this article.

**Conclusion:**

Music therapy is an important therapeutic tool for ADHD. Therefore, future research should include more robust samples and measurement scales, follow-up after experiments and further exploration of the potential connections among ADHD, music therapy, neuroimaging and neuroscientific fields.

## Introduction

Attention-deficit/hyperactivity disorder (ADHD) is one of the most prevalent mental disorders among children and is characterized by symptoms such as inattention, hyperactivity and impulsivity. ADHD affects approximately 7% of children and 5% of adolescents worldwide.^1,2^ The exact mechanism of the disorder has yet to be fully elucidated, but genetic and environmental factors play important roles.^3^ It is well established that an insufficient supply of the neurotransmitters dopamine and norepinephrine is associated with the dysfunction.^4^ For example, individuals with ADHD have higher density on the dopamine transporter (DAT) that could lead to alterations in dopamine levels in the synaptic cleft. Brain structural changes in different cortical areas, such as amygdala, frontal region and nucleus accumbens, as well as in networks related to reward processing are also observed in patients with the condition.^5^

In recent years, research on ADHD and its treatment options has increased. The treatment options for ADHD may involve medication and/or therapeutic intervention, such as music therapy, which is the use of music for clinical purposes to achieve therapeutic goals. It is evidence-based and must be conducted by a credentialed music therapist, with a variety of goals in health or education, such as reducing stress and pain, improving communication or cognitive skills and promoting wellness.^6^

Indeed, in the field of cognitive neuroscience, music is a powerful tool due to its multimodal and multisensory nature and the ability to recruit multiple cortical pathways. Scientists have associated music listening to activation in mesolimbic structures involved in reward processing including the nucleus accumbens and the ventral tegmental area.^7^ Additionally, changes in cortical regions apparently involved in reward and motivation (e.g. ventral striatum, amygdala and ventral medial prefrontal cortex) were related to pleasant experiences with music.^8^

Therefore, it seems likely that the connection between music and neuroscience can be further explored. A systematic review reported positive effects relating educational and improvisational music therapy in individuals with autism spectrum disorder (ASD).^9^ Other mental health conditions such as depression and anxiety are also promising, since a previous review of eight clinical trials reported lower levels of mood disorders after music-based sessions.^10^ Furthermore, the positive impacts of music have been shown to alter the brain activity of children with ASD, enhancing communication and social skills.^11^ These evidences suggest that the gains promoted by music-based interventions could also happen in ADHD, but this was not properly investigated.

Conversely, evidence supporting the effects of music therapy on ADHD is limited. Considering the high prevalence of ADHD and the negative effects of its symptoms, it is imperative to research new treatments for this condition. Therefore, the aim of this article was to verify whether music therapy is effective tool for alleviating ADHD symptoms among children and adolescents. Additionally, we believe this review will fill an important gap in the scientific literature by gathering and evaluating evidence and providing a synthesis of the impacts of music therapy on ADHD as summarizing the main techniques used in music therapy sessions.

## Methods

This systematic review was performed in accordance with the Preferred Reporting Items for Systematic Reviews and Meta-Analyses (PRISMA) guidelines^12^ and was conducted following an a priori established protocol (PROSPERO CRD42023474610).^13^

### Search and study selection

The PubMed, Virtual Health Library, SciELO and Cochrane Library databases were searched from inception to March 2024 without any limitations regarding the date of publication or language. The following terms were used in the search: ADHD OR attention deficit hyperactivity disorder AND music therapy. A structured search strategy based on the PICOS framework was used.^14^ Studies were included if they met the following criteria: a) the study population was children or adolescents diagnosed with ADHD meeting Diagnostic and Statistical Manual of Mental Disorders; b) the interventions involved music therapy approaches; c) the comparison was to a control group; and d) the studies were designed as control trials.

Two reviewers working independently considered the potential eligibility of each of the titles and abstracts retrieved via the search strategy. After excluding ineligible studies, the full texts of the remaining studies were screened. Then, each full-text study was assessed independently to determine study inclusion. Disagreements were resolved by consulting a third reviewer. Studies investigating the association between music therapy and the diagnosis of ADHD patients were screened. The inclusion criteria were as follows: (i) children and adolescents with symptoms of ADHD who were exposed to music therapy sessions; (ii) specific scales for the symptoms of ADHD, meeting Diagnostic and Statistical Manual of Mental Disorders; and (iii) a comparator group. Studies in which participants had other neurological, psychiatric, or learning disorders were excluded. Studies that did not evaluate ADHD symptoms as an outcome were excluded. The data were independently extracted by the reviewers and cross-checked. Any disagreements were resolved by consensus.

### Data extraction and quality assessment

Data on authors, year and publication, number of children/adolescents, age, type of intervention and main results were extracted using a standardized form. The following variables were extracted from all studies: authors, country and year of publication and patient characteristics.

The quality assessment of the individual studies was conducted using the Newcastle-Ottawa Scale. The details of the quality assessment are reported in Table 1. See Table 2 below for the list of included articles.

**Table 1:** Newcastle-Ottawa Scale to assess the quality of the included articles

Study	Selection				Comparability	Exposure			Score (0-9)
	Adequate definition of cases	Representativeness of cases	Selection of controls	Definition of controls	Study controls (most important factor or additional factor)	Ascertainment of exposure	Same method of ascertainment for cases and controls	Non-response rate	
Rickson and Watkins, 2003^15^	★	★	★	★			★	★	6-9
Rickson, 2006^16^	★	★	★		★	★	★	★	7-9
Zhu, 2022^17^	★		★		★		★	★	5-9

**Table 2:** Summary of the articles included in the systematic review

Study author, year, country, publication	Objective	Intervention	Experimental group	Control group	Age (year)	Outcome
Rickson, 2003^15^ (New Zealand) J Music Ther	To verify if MT reduces aggressive behavior	Active/receptive MT percussion, rhythm, listening, 16 ses, 2 times/week, 30-45 min	Boys with ADHD, ODD, development delayn=6 / n=5	Waitlist, same diagnosis and interventionn=4	11–15	Interaction improved. May increase disruptive behavior at school
Rickson, 2006^16^ (New Zealand) J Music Ther	To compare two MT approaches on impulsivity	8 ses of instructional: rhythm, percussion. 8 of improvisational: percussion/write songs	Boys with ADHD and comorbiditiesOn medicationn=4 / n=4	Waitlist. Same diagnosis. On medication. Received ‘Best practices’ after study.n=5	11–16	No difference. Instructional MT might reduce agitation in the classroom
Zhu, 2022^17^ (China) Psychiatr Danub	To discuss MT and CBT influence on cognitive skills	Active MT: attention, improvisation, rhythm. 45 min/ses, 1 time/week, 16 weeks. CBT	Children with ADHDn=60	Children with ADHD. Blank, experimented same therapy after trialn=60	2–7	Cognitive functions improved, ADHD symptoms alleviated

CBT = cognitive behavioural therapy; MT = music therapy; ODD = oppositional defying disorder.

### Statistical analysis

The mean change and preintervention standard deviation data were entered into SPSS version 21 to calculate the pooled standard mean difference (SMD). The SMD was selected as the appropriate measure of effect because different rating scales were used across the included trials. A random effects model was used, where measures of ADHD were analysed as continuous variables and compared between the music therapy group and the control group. A random effects model was chosen because of the anticipated heterogeneity among trials. SPSS uses Hedges’ adjusted G to calculate the SMD, which includes an adjustment for small sample bias. The I² statistic was used to analyse heterogeneity among trials. However, because this statistic is not good for detecting heterogeneity in meta-analyses involving a few trials, the p value for significance was set at 0.1.^18^ Due to the small number of trials included in this analysis, we were unable to investigate the effects of publication bias using funnel plots. It has been suggested that publication analyses should only be performed when more than 10 studies have reported a particular outcome.^19^ After data mensuration, the R software was used to calculate the statistical power of this meta-analysis.

## Results

**Figure 1:**
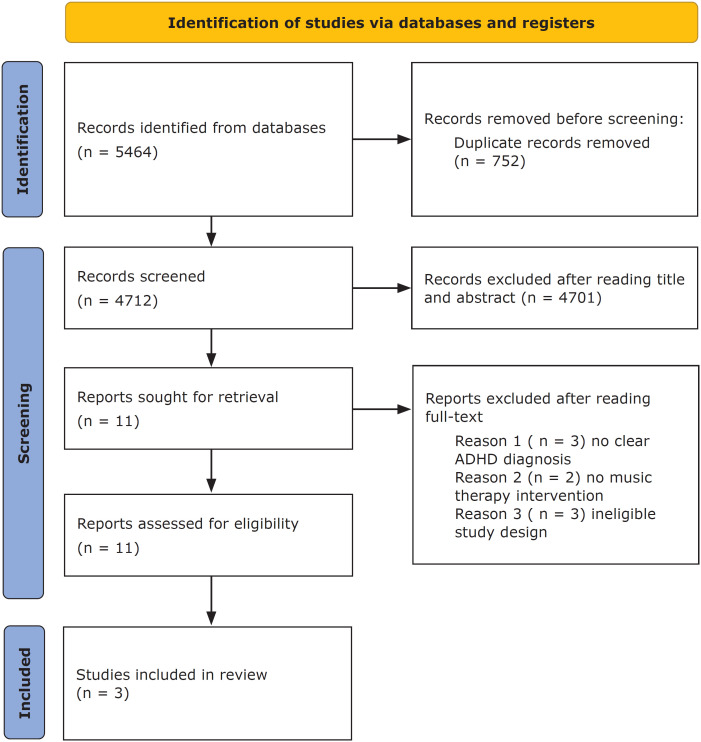
PRISMA diagram showing the process of identifying, screening and including eligible studies for this review. ADHD = attention-deficit/hyperactivity disorder.

A total of 5,464 articles were initially considered for screening. Among these, 752 duplicates were identified by manually checking the titles. After the removal of duplicates, 4,701 articles were discarded based on the reading of the titles and abstracts, and 11 studies were selected for the full reading of the texts. Then, another 8 studies were excluded because they did not meet the eligibility criteria: 3 with no music therapy (MT) intervention, 2 showed no clear ADHD diagnosis meeting Diagnostic and Statistical Manual of Mental Disorders and 3 had ineligible study designs. A PRISMA diagram of the study selection process is shown in Figure 1. Therefore, 3 articles were considered sufficiently homogeneous to be included in the meta-analysis. The results of the statistical power analysis mensuration showed that this meta-analysis has a mean power of effect size. Under this set of values, our power to detect a mean difference under a Random-Effects model is 86.62%.

The three articles included in the analysis adopted group music therapy associated with active (making music) or receptive (listening to music) approaches. The participants ranged in age and gender. Two samples^15,16^ consisted of boys with a mean age of 13 years and the other one was composed by boys and girls with ages between 2 and 7 years.^17^ All the included articles in this study followed Diagnostic and Statistical Manual of Mental Disorders. Percussion instruments, rhythmic activities and improvisational models were the most common strategies used by the therapists. Although the studies did not have the exact same design, similar results were reported, suggesting that regardless of the approach adopted, the music therapy group performed differently than the control group. Table 2 summarizes the included studies.

One study^15^ investigated the influence of music-based group sessions on 15 aggressive boys struggling with social, emotional and learning difficulties in a residential school. Students were randomly assigned to two intervention groups (group 1: n = 6, all with a formal diagnosis of ADHD and group 2: n =5, 3 in 5 with ADHD), that received the music therapy intervention during school term 3, and a waitlist control group (group 3: n = 4, 1 in 4 was diagnosed with ADHD). The control group was offered the treatment on the next school term. Half of the participants were under psychotropic medication, mostly stimulants. The authors used the Developmental Behaviour Checklist (DBC) to measure music therapy efficacy.

The findings suggest that group music therapy can promote autonomy, creativity and better relationships in boys with aggressive behavior and other disorders. On the other hand, it could lead to a disruptive behavior in classroom. More studies are recommended.

The second article analysed used two approaches of music therapy to compare its impact on motor impulsivity of 13 adolescents all diagnosed formally with ADHD (both experimental and control groups) as well as learning difficulties. Additionally, many participants had comorbidities, such as obsessive-compulsive disorder (OCD) and oppositional disorders (OD). The subjects formed two experimental groups (group B: n = 4, and group C: n = 4), that received 8 sessions each of instructional and improvisational music therapy, and a "waitlist" control group (group A: n = 5), that participated of a "best practices" treatment after the study was concluded. All adolescents were taking stimulant medication during the research. A Synchronized Tapping Task (STT) and the parent and teacher versions of Conners’ Rating Scales Restless-Impulsive (R-I) and Hyperactive-Impulsive (H-I) subscales were used to measure the impulsivity levels.

No relevant difference was found between the two models. However, the instructional approach may have contributed to decrease quietness and impulsivity of subjects in classroom.^16^

The third article^17^ included in this meta-analysis displayed a total sample of 120 children, all with a formal diagnosis of ADHD equally and randomly divided into observation and control groups, each one with 60 participants. The research objects were offered music therapy combined to cognitive behavioral (CBT), while the control was a blank group that received the same intervention after the experiment.

Improved cognitive functions, learning skills, impulsivity and hyperactivity of children diagnosed with ADHD were observed. Group MT sessions were offered for 16 weeks and included attention training, improvised playing and echo playing among other strategies. The following scales were applied before and after the experiments: ADHD rating scale for parents; numerical cross-attention test (NCT); Wisconsin card sorting test (WCST); Combined Raven's Test (CRT); Wechsler intelligence scale for children (WISC), fifth edition; Concentration/attention factor (C factor); and Conner's Children Behaviour Scale.

**Figure 2:**
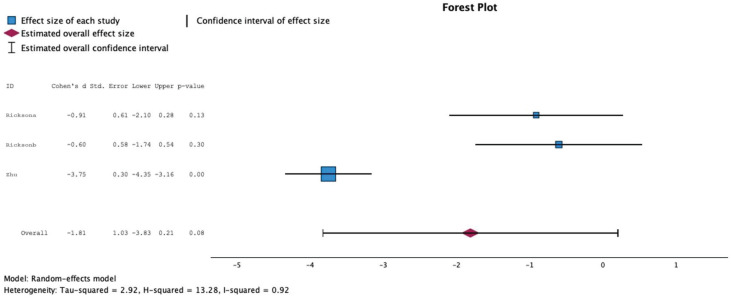
Forest plot for the included studies.

A meta-analysis of the three studies involving 148 participants (79 ADHD patients and 69 controls) evaluated the efficacy of music therapy concerning hyperactivity and impulsivity symptoms. The results showed an improvement in ADHD patients compared to controls (effect size: 1.18; CI: −3.8 – 0.21; p = 0.08; Figure 2). In addition, a significant amount of heterogeneity among trials was also found (I2 = 92 %). Publication bias was not assessed because there were inadequate numbers of included studies to properly assess a funnel plot or to perform more advanced regression-based assessments.

## Discussion

This is the first meta-analysis to evaluate music therapy as a tool for the treatment of children and adolescents with ADHD. Although the main findings of our meta-analysis have showed a trend for the efficacy of music therapy in ADHD symptoms, more studies need to investigate the music therapy role on ADHD.

Regarding hyperactivity/impulsivity and music therapy, studies have revealed improvements in restless behaviour and impulse control through structured rhythmic activities and percussion. Two contrasting music therapy approaches were employed to measure the impacts of music on motor impulsivity. The improvisational group (humanistic approach, social interaction-centred sessions) did not show a statistically significant difference from the instructional group (more structured and active therapy). However, the second model might have helped to reduce restlessness and impulsivity in the classroom.^16^ Recent research has also shown that percussion instruments, such as drum training, improve the brain functioning, stimulation and communication of people living with autism. In addition, it could also be applied to ADHD patients struggling with inhibitory control and attention difficulties.^20^

Regarding attention abilities, a study involving time processing and discrimination of simple sounds and music using a keyboard showed a positive modulation of inattention symptoms. The findings describe that counting sound events requires a high attentional demand.^21^ Additionally, an experiment involving music and ADHD reported that these individuals show time distortions and difficulties in keeping the beat to music or a metronome, for example. Thus, these findings could suggest that music and sound, time processing, attentional abilities and impulsive behaviours are closely related in individuals with ADHD.^22^

Another benefit observed after music-based treatment is youth community relations. Groups can promote autonomy and creativity in aggressive youth, improving peer relations. On the other hand, disruptive behaviour in the classroom might be expressed if sessions are not highly structured. Combined with CBT, music sessions improved social skills, such as relationships with peers, self-management, following instructions and eye contact.^15,23^

Children with ADHD experienced enhancements in learning difficulties, behaviour problems, impulsivity and hyperactivity after continuous attention and rhythmic training and with CBT.^17^ Previous research corroborated these findings, showing that musical training balanced brain development deficits, including learning difficulties, e.g., dyslexia.^24^

Researchers found^25^ that 36 children and adolescents with ADHD and comorbid depression received music therapy which led to the activation of serotonin and improved stress coping skills based on measurements of 5-HT, cortisol, blood pressure and heart rate. In line with these findings, music could be an alternative for investigating the neural basis of anhedonia (loss of pleasure in daily activities) in individuals with various psychiatric conditions, such as depression, schizophrenia and bipolar disorder.^7^

From a neuroscientific perspective, it is well established that music stimulates different cortical regions. These sounds can be related to an increase in mesolimbic system activity, which is crucial for reward mechanisms, along with the ventral striatum, nucleus accumbens, cerebellum, insula, mesencephalon, thalamus, anterior cingulate cortex and orbitofrontal cortex. Some of these regions are altered in individuals with ADHD.^26,27^

Despite these findings, the lack of a large body of literature relating music therapy, ADHD and brain imaging is a limitation of this study. A second limitation is that inattention, which is a core symptom of the disorder, have not received due attention, since the three articles included focused mainly on hyperactivity and impulsivity outcomes.

## Conclusions

As demonstrated by the studies found in this systematic review and meta-analysis, music therapy is an important therapeutic tool for children and adolescents with ADHD. Outcomes were especially associated with a trend of efficacy regarding hyperactive and impulsive symptoms in individuals with the disorder.

Therefore, further studies are suggested, including more robust samples and measurement scales, follow-up after experiments and further exploration of the potential connections among ADHD, music therapy, neuroimaging and neuroscientific fields.

## Data Availability

The data that support this study are available from the authors upon request.

## References

[1] (c-2025). American Psychiatric Association [Internet].

[2] Salari N, Hooman G, Nasrin A, Adibeh R, Mohammad HS, Amir HH (2023). The global prevalence of ADHD in children and adolescents: a systematic review and meta-analysis. Ital J Pediatr.

[3] Curatolo P, D’Agati E, Moavero R (2010). The neurobiological basis of ADHD. Ital J Pediatr.

[4] Signor R, Santana AP (2020). The constitution of the subjectivity of the child diagnosed with attention deficit hyperactivity disorder. Bakhtiniana.

[5] Silva BS, Grevet EH, Silva LCF, Ramos JKN, Rovaris DL, Bau CHD (2023). An overview on neurobiology and therapeutics of attention-deficit/hyperactivity disorder. Discov Ment Health.

[6] American Music Therapy Association [Internet] Silver Spring: about music therapy: what is music therapy; c1995-2016.

[7] Menon V, Levitin DJ (2005). The rewards of music listening: Response and physiological connectivity of the mesolimbic system. Neuroimage.

[8] Blood AJ, Zatorre RJ (2001). Intensely pleasurable responses to music correlate with activity in brain regions implicated in reward and emotion. Proc Natl Acad Sci USA.

[9] Mayer-Benarous H, Benarous X, Vonthron F, Cohen D (2021). Music therapy for children with autistic spectrum disorder and/or other neurodevelopmental disorders: a systematic review. Front Psychiatry.

[10] Ibiapina ARS, Lopes-Junior LC, Veloso LUPV, Costa APC, Silva FJG, Sales JCS (2022). Effects of music therapy on anxiety and depression symptoms in adults diagnosed with mental disorders: a systematic review. Acta Paul Enferm.

[11] Sharda M, Tuerk C, Chowdhur R, Kevin J, Foster N, Custo-Blanch M (2018). Music improves social communication and auditory–motor connectivity in children with autism. Transl Psychiatry.

[12] Page MJ, McKenzie JE, Bossuyt PM, Boutron I, Hoffmann TC, Mulrow CD (2021). The PRISMA 2020 statement: an updated guideline for reporting systematic reviews. BMJ.

[13] PROSPERO (2023). Systematic review protocol: PROSPERO international prospective register of systematic reviews.

[14] Richardson WS, Wilson MC, Nishikawa J, Hayward RS (1995). The well-built clinical question: a key to evidence-based decisions. ACP J Club.

[15] Rickson DJ, Watkins WG (2003). Music therapy to promote prosocial behaviors in aggressive adolescent boys--a pilot study. J Music Ther.

[16] Rickson DJ (2006). Instructional and improvisational models of music therapy with adolescents who have attention deficit hyperactivity disorder (ADHD): a comparison of the effects on motor impulsivity. J Music Ther.

[17] Zhu C (2022). Effects of music therapy combined with cognitive behavioral intervention on the cognitive ability of children with attention deficit hyperactivity disorder. Psychiatr Danub.

[18] Higgins JPT, Thomas J, Chandler J, Cumpston M, Li T, Page MJ (2019). Cochrane Handbook for Systematic Reviews of Interventions.

[19] Sterne JAC, Sutton AJ, Ioannidis JPA, Terrin N, Jones DR, Lau J (2011). Recommendations for examining and interpreting funnel plot asymmetry in meta-analyses of randomised controlled trials. BMJ.

[20] Cahart MS, Amad A, Draper SB, Lowry RG, Marino L, Carey C (2022). The effect of learning to drum on behavior and brain function in autistic adolescents. Proc Natl Acad Sci USA.

[21] Carrer LRJ (2015). Music and sound in time processing of children with ADHD. Front Psychiatry.

[22] Puyjarinet F, Bégel V, Lopes R, Dellacherie D, Dalla Bella S (2017). Children and adults with attention-deficit/hyperactivity disorder cannot move to the beat. Sci Rep.

[23] Gooding LF (2011). The effect of a music therapy social skills training program on improving social competence in children and adolescents with social skills deficits. J Music Ther.

[24] Serrallach B, Groß C, Bernhofs V, Engelmann D, Benner J, Gündert N (2016). Neural biomarkers for dyslexia, ADHD, and ADD in the auditory cortex of children. Front Neurosci.

[25] Park JI, Lee IH, Lee SJ (2023). Effects of music therapy as an alternative treatment on depression in children and adolescents with ADHD by activating serotonin and improving stress coping ability. BMC Complement Med Ther.

[26] Chatterjee D, Hegde S, Thaut, M (2021). Neural plasticity: The substratum of music-based interventions in neurorehabilitation. NeuroRehabilitation.

[27] Faraone SV (2018). The pharmacology of amphetamine and methylphenidate: relevance to the neurobiology of attention-deficit/hyperactivity disorder and other psychiatric comorbidities. Neurosci Biobehav Rev.

